# Impact of an intensive outpatient rehabilitation on non-motor patients’ reported outcomes in PD: the INTENSO study

**DOI:** 10.1038/s41531-025-01035-7

**Published:** 2025-06-20

**Authors:** Marianna Capecci, Nicolò Baldini, Elisa Andrenelli, Alice Lambertucci, Paola Bisoglio, Martina Grugnetti, Hibel Margherita, Maria Gabriella Ceravolo

**Affiliations:** 1 Department of Experimental and Clinical Medicine, Politecnica delle Marche University, Ancona, Italy; 2 Neurorehabilitation Clinic, Marche University Hospital, Ancona, Italy

**Keywords:** Neurological manifestations, Movement disorders, Parkinson's disease

## Abstract

Non-motor symptoms in Parkinson’s disease (PD) can reduce quality of life and increase disability. This historical cohort study investigated how rehabilitation intensity influences non-motor symptoms. The primary outcomes were changes in non-motor symptoms in the short and medium term. Secondary outcomes were changes in disability burden, motor symptom severity, and freezing of gait after treatment. Measurements were taken before (T0) and after treatment (T1) and 6 ± 1 months after T1 (T2). According to total training duration, 24 patients with PD were assigned to High-Intensity Training group (HIT, 1800 min) and 24 to Low-Intensity Training (LIT, less than 900 minutes). At T1, only the HIT group showed clinically significant improvements in non-motor symptoms, which were maintained at T2. In contrast, the LIT group experienced worsening disability at follow-up. Multivariate analysis revealed training intensity and baseline disability as predictors of improvement. These findings support the benefits of high intensity exercise in PD management.

## Introduction

Parkinson’s Disease (PD) is a chronic progressive neurodegenerative disease characterised by motor and non-motor symptoms. The latter include cognitive, behavioural, and autonomic disorders. It has been observed that the prevalence of certain domains has increased over time. These domains include sleep quality, gastrointestinal symptoms, cognition, and skin issues such as hyperhidrosis and seborrhea. Non-motor symptoms strongly affect psychological, physical, and social functions, largely leading to a reduction in the patient’s quality of life and an increase in disability^1^. However, effective treatments are still a long way off. The National Institute for Health and Care Excellence (NICE) has published guidelines on managing Parkinson’s disease in adults, including recommendations for treating non-motor symptoms such as sleep disorders. Levodopa or a dopamine agonist can be used to treat sleep disorders, with moderate supporting evidence. Clonazepam and melatonin can help to control REM behavioural disorders. For psychosis-related symptoms, NICE guidelines recommend medications such as quetiapine and clozapine, despite the low level of evidence^2^. The Movement Disorders Society reviewed level I studies and found clinically useful interventions for depressive symptoms, such as pramipexole or venlafaxine; dementia, such as rivastigmine; sexual dysfunction, such as sildenafil; and drooling, such as botulinum toxin type A and B injections^2–5^. However, significant progress is still needed before effective pharmacological treatments become available for the full spectrum of non-motor symptoms. Therefore, non-pharmacological treatments might be successful in reducing their impact. The road ahead is still challenging, with most treatments still in the investigational phase, despite none of them reaching a high level of recommendation. However, promising findings are emerging from the use of transcranial magnetic stimulation to manage depression or mild cognitive impairment, and acupuncture to deal with fatigue^3^. A higher level of evidence was found for cognitive behavioral treatment for depression and impulse control disorder^3^. Over the years, attention to this topic has grown, and several studies have investigated the role of exercise in PD. One of the first aspects to be explored was its possible neuroprotective effect. Mechanisms activated by the exercise include angiogenesis, neuroplasticity, neuroprotection, neurogenesis, anti-inflammatory effects, improvement of mitochondrial function and reduced oxidative stress, increase in neurotrophic factors, and restoration of the basal ganglia circuits^6–10^. The beneficial effects of most types of exercise on Parkinson’s disease have been demonstrated in the literature. For instance, aqua-based exercises have shown a large beneficial effect on quality of life and a moderate effect on the severity of motor symptoms. Furthermore, dance, gait/balance/functional training, and multidomain interventions demonstrate moderate effects on motor symptom severity, while endurance training has shown a moderate impact on quality of life^11^. Progressive resistance exercises improve muscle strength, functional outcomes, and non-motor symptoms^12^; aerobic exercises can not only impact non-motor domains (e.g., attention, problem-solving abilities, and verbal fluency) but also reduce depression^13^. Several studies investigated the role of exercise intensity. A phase II randomised clinical trial (RCT) found that high-intensity training is feasible and safe in PD and has a positive outcome on more motor domains than low-intensity training^14^, in line with the meta-analysis by Uhrbrand et al.^15^ Another phase II RCT hypothesized that a high-intensity exercise slowed down PD progression and provided the rationale for the phase III clinical trial – SPARX3 that is still ongoing^16–18^. Few other authors focused on the benefits of exercise training on non-motor symptoms^19^. Evidence of efficacy is emerging but is limited by the small study samples, the heterogeneity of the rehabilitation approaches, and the diversity of outcomes selected (i.e., depression, anxiety, sleep disorders, or cognitive disorders)^19^.

Considering the acknowledged superiority of high-intensity task-oriented exercise on motor symptoms^6–10,20,21^, we sought to investigate whether and to what extent this approach would also improve non-motor patient-reported outcomes, more than low-intensity exercise.

The study aims to evaluate the impact of different intensity rehabilitation protocols on the short and medium-term severity of non-motor disorders in patients with PD.

## Results

**Fig. 1:**
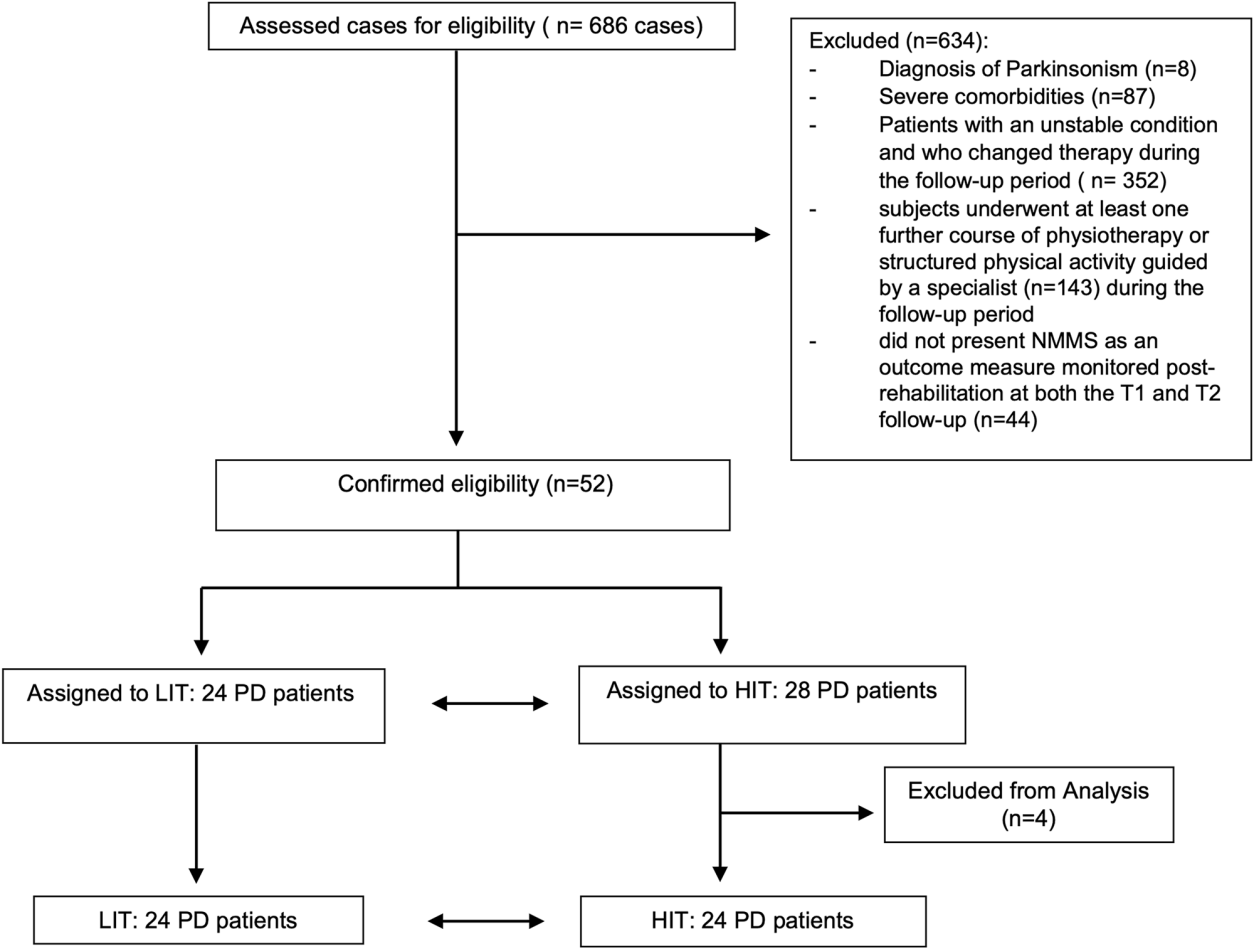
Study flowchart. The complete flow of patients through the study conforms to the STROBE (Strengthening the Reporting of Observational Studies in Epidemiology) requirements. HIT High Intensity Training, LIT Low Intensity Training.

Out of 686 cases consecutively referred to our rehabilitation facilities, 96 met the inclusion criteria, but only 52 had complete documentation of the main outcome measure at all time points. Based on the training modality, 28 were assigned to the HIT group and 24 to the LIT. Four patients were excluded from the HIT group following the propensity score matching. The complete flow of patients through the study conforms to the STROBE (Strengthening the Reporting of Observational Studies in Epidemiology) requirements^22^, and is displayed in Fig. 1.

### Baseline demographic and clinical characteristics

As described in Table 1, there were no statistically significant group differences in demographic and clinical features at baseline. The average training intensity for the LIT group was 612 minutes (range: 450–900 minutes), whereas the HIT group received 1800 min of total training. No changes in the pharmacological or non-pharmacological treatments were observed across the study in any subject. Antidepressants, with or without benzodiazepines, to improve sleep quality, were used by 45% of participants in the HIT group and 55% in the LIT group. Drug regimens remained stable during the six-month study period.

**Table 1:** Demographic and clinical data at baseline

Parameter		HIT n. 24 (11 ♂; 13 ♀)	LIT n. 24 (13 ♂; 11 ♀)	Group comparison at t0
**AGE (years)**	Mean (SD)	67.3 (6.7)	69.6 (6.3)	0.278
	Min-max	55–78	56–81	
**Disease duration**	Mean (SD)	8.5 (6.4)	11.5 (7)	0.114
	Min-max	3–26	3–23	
**Hoehn and Yahr stage (modified)**	Median (Q1; Q3)	2 (2;3)	2 (2;4)	0.04
	Min-max	2–4	1–4	
**Rehabilitation intensity (minutes)**	Mean (SD)	1800	612.5 (182)	**<0.001**
	Min-max	1800	450-900	
**NMSS**	Mean (SD)	38.75 (15.1)	43.3 (29.4)	0.749
	Min-max	17-79	5-101	
**MDS-UPDRS I**	Mean (SD)	12.9 (3.6)	12.2 (6.0)	0.513
	Min-max	7–19	3-23	
**MDS-UPDRS II**	Mean (SD)	13.1 (5.9)	14.9 (6.7)	0.226
	Min-max	7–29	2-28	
**MDS-UPDRS III**	Mean (SD)	21.3 (11.5)	20.0 (8.4)	0.885
	Min-max	4–47	7-37	
**MDS-UPDRS IV**	Mean (SD)	3.9 (3.4)	3.8 (4.1)	0.723
	Min-max	0–11	0-17	
	HIT n. 24	LIT n. 18		
**FOG-Q**	Mean (SD)	10.3 (5.6)	10.1 (7.3)	0.909
	Min-max	0–17	0-22	

*HIT* High Intensive Training, *LIT* Low Intensive Training, *NMSS* Non-Motor Symptoms Scale, *MDS - MDS-UPDRS* Movement Disorder Society - Unified PD Rating Scale; I = Non-motor symptoms; II = Motor Experiences of Daily Living; III = Motor Examination; IV = Motor Complications, *FOG-Q* Freezing of Gait- questionnaire. Statistically significant values (*p* < *0.01*) are presented in bold.

### Impact of exercise intensity on clinical and functional outcomes over time

Table 2 shows the pre-post-treatment evolution of all the outcome measures and the within and between-group comparisons. NMSS and MDS-UPDRS I scores improved at T1, only in the HIT group. A statistically significant within-group change was observed for MDS-UPDRS I only. The average change exceeded the Minimal Clinically Important Difference (MCID) for the MDS-UPDRS I [33,34]. At T2, the MDS-UPDRS I worsened in all subjects more than the MCID, whereas the NMSS score significantly worsened only in the LIT group. We did not find significant trends in the subitem scores of the NMSS or any subgroup. The MDS-UPDRS II and III subscores and the FoGQ score improved at T1 in both groups, though only at a significant level in the HIT subgroup, where the changes exceeded the MCID. At T2, the improvement in secondary outcome measures was maintained in the HIT group despite a slight trend to baseline values. The MDS-UPDRS II score at T2 was significantly better in HIT than LIT subjects, who exhibited a clinically meaningful worsening.

**Table 2:** Pre-post treatment outcomes’ evolution and the within and between-group comparisons

	HIT				LIT			Group effect; Z-value; *p-*value
Parameters	Time	Mean (SD)	Range (Min-Max)	Time effect (vs T0); Z-value; *p-*value	Mean (SD)	Range (Min-Max)	Time effect (vs T0); Z-value; *p*-value	
**NMSS**	T0	38.8 (15.1)	17–79		43.3 (29.4)	5–101		0.32; 0.74
	T1	34.4 (17.2)	3–70	2.2; 0.03	45.5 (32.8)	7–130	1.1; 0.28	0.82; 0.41
	T2	42.2 (22.5)	18–105	1.1.; 0.27	52.6 (36.2)	12–134	**2.9; 0.003**	0.54; 0.58
**MDS-UPDRS I**	T0	12.9 (3.6)	7–19		12.2 (6.0)	3–23		0.65; 0.52
	T1	10.2 (3.9)	2–18	3.7; **0.0003**	12.3 (6.8)	2–24	0.38; 0.7	1.1; 0.26
	T2	12.6 (3.6)	7–19	0.21; 0.83	15.1 (12.2)	4–66	1.5; 0.12	0.16; 0.88
**MDS-UPDRS II**	T0	13.1 (5.9)	7–29		14.9 (6.7)	2–28		1.2; 0.23
	T1	10.1 (6.1)	2–27	**3.2; 0.0012**	13.6 (6.7)	5–23	1.4;0.16	1.5; 0.13
	T2	10.3 (6.7)	1–29	3.2; **0.0014**	15.6 (6.5)	5–26	1.2;0.23	**2.7; 0.007**
**MDS-UPDRS III**	T0	21.3 (11.5)	4–47		20.0 (8.4)	7–37		0.08; 0.94
	T1	16.2 (9.8)	2–34	3.0; **0.003**	16.1 (7.3)	6–30	0.65;0.51	0.08; 0.94
	T2	17.1 (9.9)	3–44	2.3; 0.02	18.9 (8.8)	6–35	0.53;0.59	0.78; 0.43
**MDS-UPDRS IV**	T0	3.9 (3.4)	0–11		3.8 (4.1)	0–17		0.35; 0.72
	T1	2.8 (2.5)	0–8	2.2; 0.03	1.8 (1.8)	0-5	2.0;0.04	0.31; 1.03
	T2	3.6 (2.6)	0–9	0.57; 0.57	2.6 (2.8)	0-9	1.6;0.1	1.5; 0.13
**MDS-UPDRS TOT**	T0	41.6 (16.5)	23–89		42.3 (17.7)	11–74		0.31; 0.75
	T1	31.5 (16.2)	6–67	3.6; **0.0003**	34.2 (14.7)	12–56	1.5;0.11	0.5; 0.61
	T2	31.9 (18.4)	2–80	3.4; **0.0006**	40.4 (16.3)	12–67	0.88;0.37	1.7; 0.1
**FOG-Q**	T0	10.3 (5.6)	0–17		10.1 (7.3)	0–22		0.11; 0.9
	T1	8.2 (5.1)	0–15	2.6; **0.0075**	9.5 (6.9)	0–21	0.59;0.56	0.41; 0.68
	T2	7.5 (5.3)	0–15	3.2; **0.001**	10.0 (7.4)	0–22	0.17;0.86	0.1; 0.24

*HIT* High-Intensity Training, *LIT* Low-Intensity Training, *NMSS* Non-Motor Symptoms Scale, *MDS - MDS-UPDRS* Movement Disorder Society - Unified PD Rating Scale; I Non-Motor symptoms; II Motor Experiences of Daily Living; III Motor Examination; IV Motor Complications, *FOG-Q* Freezing of Gait- questionnaire. Statistically significant values (*p* < *0.01*) are presented in bold.

NMSS and MDS-UPDRS part-I Δ values were significantly higher in HIT than LIT subjects at T1 and, concerning NMSS only, at T2 approached statistical significance (Table 3). The T1 Δ value was directly related to the total training time, both for NMSS (R^2^:17%; Std.Coeff.:0.14; F:9.3; *p*:0.004) and MDS-UPDRS I (R^2^:17%; Std.Coeff.: 13.8; F:9.3; *p*:0.004). Conversely, we did not find any role played by age or disease duration at impacting NMSS or MDS-UPDRS I change after treatment. The T2 NMSS Δ value was also directly influenced by total training time (R^2^:16%; Std. Coeff.:63.5; F:8.4; *p*:0.006).

**Table 3:** Delta (Δ) values between T1 or T2 versus T0 and intergroup comparison

Effectiveness(Δ)	GROUP	Comparison VS T0	Δ Mean (SD)	Min; Max	Inter group comparison (Z and *p-* value)
**NMSS**	HIT	T1	−0.22 (0.22)	−0.75; 0.08	−**3.04; 0.002**
	LIT	T1	0.01 (0.33)	−0.71; 0.67	
	HIT	T2	9.66 (32.79)	−60.42; 66.67	−2.1; 0.04
	LIT	T2	43.82 (53.10)	−27.03; 180	
**MDS-UPDRS PART I**	HIT	T1	−21.45(21.69)	−75; 8.33	−**3.1; 0.002**
	LIT	T1	0.73 (33.23)	−71.43; 66.67	
	HIT	T2	1.39 (45.01)	−114.12; 100	−0.5; 0.61
	LIT	T2	3.05 (40.40)	−100; 80	

*HIT* High-Intensity Training, *LIT* Low-Intensity Training; *NMSS* Non-Motor Symptoms Scale, *MDS - MDS-UPDRS* Movement Disorder Society - Unified PD Rating Scale; I = Non-Motor symptoms. Statistically significant values (*p* < *0.01*) are presented in bold.

### Identification of independent modulating factors

The multivariate analysis (Table 4) indicated total training duration and baseline MDS-UPDRS II score as independent factors of NMSS Δ value at T1 (R2:29%; F:3.4; *p* = 0.01): the higher the treatment intensity, the greater the improvement of non-motor symptoms. Conversely, the greater the activity limitation at baseline, the lower the impact of treatment on non-motor symptoms. Training intensity also directly influenced the MDS-UPDRS part I Δ value at T1. Analysis of the individual NMSS sub-items did not reveal any correlation with the examined dependent variable.

**Table 4:** Multivariate analysis

Dependent Variable	Independent variables	Std. Coeff.	T-value	*p-*value	R^2^	F-value	*p*-value
**NMSS Δ score at T1**					0.288	3.398	0.0115
	Age	−0.12	−0.82	0.413			
	Total minutes of training	−0.39	−2.9	**0.005**			
	Disease duration	−0.11	−0.84	0.401			
	NMSS T0	0.017	0.11	0.910			
	MDS-UPDRS II T0	0.355	2.555	0.014			
**NMSS Δ score at T2**					0.343	4.383	0.0027
	Age	−0.18	−1.30	0.199			
	Total minutes of training	−0.44	−3.43	**0.001**			
	Disease duration	−0.09	−0.69	0.488			
	NMSS T0	−0.34	−2.43	0.019			
	MDS-UPDRS II T0	0.006	0.044	0.965			
**MDS-UPDRS I Δ score at T1**					0.292	3.468	0.010
	Age	−0.9	−0.62	0.534			
	Total minutes of training	−0.38	−2.76	**0.008**			
	Disease duration	−0.11	−0.84	0.405			
	NMSS T0	0.38	2.630	0.011			
	MDS-UPDRS II T0	−0.07	−0.51	0.610			
**MDS-UPDRS I Δ score at T2**					0.119	1.133	0.357
	Age	0.17	1.06	0.294			
	Total minutes of training	−0.09	−0.60	0.551			
	Disease duration	0.006	0.041	0.967			
	NMSS T0	−0.28	−1.78	0.081			
	MDS-UPDRS II T0	−0.11	−0.69	0.492			

*NMSS* Non-Motor Symptoms Scale, *MDS-UPDRS* Movement Disorder Society - Unified PD Rating Scale; I = Non-Motor symptoms. Statistically significant values (*p* < *0.01*) are presented in bold.

## Discussion

The results of our single-centre historical cohort study provide insights into the potential benefits of exercise in improving non-motor symptoms in PD patients. The findings are relevant as few options exist for treating such symptoms, significantly impacting quality of life^23^. In this study, a multimodal and intensive approach effectively reduced the disease burden due to non-motor symptoms.

According to existing literature, regular exercise and physical activity of any type are essential to lower the severity of motor symptoms and prevent disability progression^11^. Studies and guidelines agree that people with Parkinson’s Disease (pwPD) should be encouraged to adhere to an active lifestyle and carry on personalized physical training. Aerobic exercise of different intensities is recommended to impact the performance of pwPD, increase BDNF production^24,25^, and improve cognition. Training protocols should consider the diversification of tasks and the repetition of gestures to enhance motor learning^26^ and the tailoring of exercises to pwPD capacities to ensure patients’ adherence^27^.

Exercise also positively affects non-motor symptoms^12,13^. Our findings are consistent and expand such evidence, looking into the components of training that most impact non-motor symptoms. By dividing the study sample into two subgroups based on exposure to different training schedules, we sought to investigate the role of exercise intensity. We found a positive impact of high-intensity training on non-motor symptoms in the short and medium term.

Few studies compare the effect of low to high-intensity training in pwPD and mainly investigate the outcome in terms of motor symptoms^17,28,29^. Our results indicate that high-intensity exercise training is safe and feasible and leads to overall improvement in patients’ condition: motor and non-motor symptoms, disability burden, and related PD complications such as FOG. At variance with previous investigations of the relationship between exercise and non-motor symptoms^14,28,30^, we studied a heterogeneous sample of people with PD, also including those in advanced phase (the Hoehn and Yahr stage ranged from 1 to 4), evenly distributed across the two subgroups. This is a turning point, as even if non-motor symptoms occur in all disease stages, they become more disabling in the later ones^31^. We observed an improvement in non-motor symptoms, exceeding the minimum clinically important difference for MDS-UPDRS I, only in the HIT group, which maintained such benefit at T2. Conversely, non-motor symptoms significantly worsened in the LIT group at T2. The total training duration was the main independent factor of a positive change in non-motor symptom scale scores at any time point. Hence, modulating the exercise intensity by increasing the single session duration impacts patients’ health not only in the short term, as suggested by Abruzzese et al.^32^, but also in the medium term. i.e., up to six months after the end of training.

The biological rationale underlying the effects of exercise is ascribed to the release of monoamines^33,34^ and endorphins^35,36^. While these molecules are indicated as the main factors of the positive effects of exercise on mood^37^, the exercise-driven release of brain-derived growth factor (BDNF) not only affects mood^38^ but also triggers neuroplasticity, making the brain more receptive to learning and consolidating new information^39^. All the quoted mechanisms may lead to an improvement in non-motor symptom scale scores that rate both mood and cognition.

Intensive training in the HIT group was also the main factor in improving secondary endpoints (i.e., independence in ADL, motor symptoms, and freezing of gait). Independence in ADL can be directly impacted by longer exposure to occupational therapy and, indirectly, by increased endurance (targeted by aerobic training). Among motor symptoms, exercise is expected to impact axial functions (trunk posture, transfer and walking capacity) more than segmental aspects (tremor, rigidity and hypokinesia), which are better addressed by antiparkinsonian drugs. In particular, FOG is a tricky and challenging feature that is adversely influenced by non-motor functions, like attention, impulsivity, and anxiety, and positively affected by cueing strategies and dual-task practice. Our findings show that high-intensity training has a statistically significant impact on FOG in the short and medium term. The role of FOG-directed training is endorsed by the current literature, with inconsistent data regarding the carry-over effects after the end of the training^40,41^.

In summary, designing a high-intensity multimodal treatment combining aerobic exercise with occupational therapy and dual-task training may be the key to success in the rehabilitation of people with PD.

In this study, the multivariate analysis also pointed to the independence of ADL as a modifier of the relationship between exercise and non-motor symptoms. This finding underscores the role of a timely start of high-intensity training^42,43^ that should be offered to pwPD since the very early disease phases to maximise the effectiveness and sustainability of its effects.

Due to the retrospective design and the small sample size, we acknowledge the study limitations. To obtain a reasonably informative sample and fill the gaps in clinical records, we screened an extensive dataset and filtered only the subjects with complete data. The small sample did not allow us to determine which non-motor symptoms, among those rated by the NMSS or MDS-UPDRS part I, benefited most from different training intensities. A multicenter prospective study is warranted to provide more solid evidence on a larger sample and check the effects of high-intensity training on non-motor symptoms in PD.

In conclusion, this historical cohort study supports the potential benefits of exercise, particularly high-intensity training, in improving both motor and non-motor symptoms in people with PD. The positive outcomes observed and the potential to slow down the disease-related disability progression are encouraging. These findings highlight the need for further investigation, enhancing the reliability of the results, with the scope of developing effective rehabilitation strategies that can be integrated with pharmacological therapy to optimize the management of the disease and improve patients’ health.

## Methods

### Study design

Single-center historical cohort study of people with PD consecutively referred to the outpatient rehabilitation facility of a Center for Diagnosis and Treatment of Movement Disorders, based in a university hospital in Italy.

The study used data collected retrospectively from patients’ records. The data were pseudo-anonymized so that each subject was matched to an alphanumeric code, and his/her demographic and clinical information were added to an Excel sheet for subsequent analysis.

### Subject selection

We extracted PD cases from medical records based on the following eligibility criteria. Inclusion criteria: adults diagnosed with Idiopathic PD, Hoehn & Yahr stage ≥ 1 < 5, consecutive referral to the rehabilitation facility between 1 January 2014 and 31 December 2019 to receive outpatient physiotherapy treatment for the management of motor disability, specifically targeting gait, balance, or posture disorders; completion of the course of rehabilitation treatment; availability of Non-Motor Symptom Scale (NMSS)^44^ and Unified Parkinson’s Disease Rating Scale (MDS-UPDRS)^45^, recorded before (T0) and after (T1) treatment and 6 plus or minus 1 months later (T2). Exclusion criteria: diagnosis of any primary or secondary parkinsonism rather than PD, symptom onset since less than three years, any concomitant neurological disease (e.g. polyneuropathy, stroke, etc); any other chronically disabling disease (e.g., severe heart, liver or kidney failure, cancer, psychiatric disorders, limb amputation, severe musculoskeletal or neuropathic pain); changes in antiparkinsonian drug therapy during the whole study period, particularly with regard to pharmacological or non-pharmacological interventions for non-motor symptoms; exposure to a course of physiotherapy during the 6 months preceding the study.

The model of care applied at this Center for Diagnosis and Treatment of Movement Disorders is described in Capecci et al.^46^. In a multidisciplinary team, the neurologist and physiatrist assess PD-related motor and non-motor symptoms through a standard approach, including the MDS-UPDRS and NMSS scoring. They also evaluate disease-related disability and pose indications for rehabilitation. The individual rehabilitation plan is discussed with experienced physical therapists who initiate outpatient treatment if the patient resides in the catchment area. Based in a university hospital, the staff is regularly engaged in clinical research programs that require the administration or validation of standard assessment and treatment procedures. Inter-rater reliability is verified by Cohen’s K-value ≥ 0.8. In particular, the assessment protocol for which the inter-rater reliability was checked for patients with PD included MDS-UPDRS, NMSS, and Freezing Of Gait Questionnaire (FOG-Q)^47^.

### Interventions

In line with international guidelines^27,48–50^ and based on the rationale^11,20,21,26^ for training in people with chronic neurodegenerative disorders, rehabilitation provided to PD patients in our rehabilitation facility routinely includes both aerobic and task-oriented training.

In our clinical practice, each outpatient session typically lasts between 60 and 90 minutes, with 10 to 20 sessions per cycle and a frequency ranging from 2 to 3 sessions per week. As a result, the Low intensity group received 10 sessions of 60 to 90 minute duration, twice per week, for a total of 5 weeks; the High intensity group received 20 sessions of 90 minute duration, 3 times per week, for a maximum of 7 weeks.

Irrespective of total course intensity, each session comprises at least 15 minutes of aerobic training (over ground or treadmill) and 10 minutes of flexibility and strengthening exercise.

No less than 10 minutes of balance training, 10 minutes of overground training in dual tasks, and 15 minutes of occupational therapy are additionally delivered. Table 5 provides a comprehensive overview of the structure of the exercise programme. The duration of the single training components in each session may increase according to the patients’ functioning profiles, requesting a more intensive practice of gait, balance, or trunk alignment or a focused training of arm dexterity or basic ADL.

**Table 5:** Comprehensive overview of the structure of the exercise programme

Component	Minimum Duration	Possible Interventions
Aerobic training	15 minutes	- Overground gait training - Treadmill gait training
Flexibility & strengthening	10 minutes	- Stretching for trunk mobility - Joint mobility, focusing first on multiple and then on single joints. - Strengthening of the knee and hip extensors - Strengthening ankle plantar flexors
Balance training	10 minutes	- Balance training (with or without non immersive Virtual Reality balance boards)
Dual task overground training	10 minutes	- Functional gait training combined with verbal, visual or motor tasks. - Cues based training
Occupational therapy	15 minutes	- Training of ADLs - Training of iADLS.

*ADL* Activity of Daily Living, *iADL* instrumental Activity of Daily Living.

Based on the total amount of training performed during one rehabilitation cycle, two different rehabilitation protocols could be identified:

- High-Intensity Training (HIT): patients in this group received 1800 min of training globally.
- Low-Intensity Training (LIT): patients received less than 900 minutes of training.

### Study endpoints

The primary endpoints were changes in non-motor symptoms measured by NMSS and MDS-UPDRS part I after treatment in subgroups exposed to different training intensity.

Secondary endpoints were changes in PD-related disability measured by MDS-UPDRS part II, motor symptoms severity assessed by MDS-UPDRS part III, and Freezing of Gait by FOG-Q after treatment.

As per the inclusion criterion, the outcome measures had to be collected before treatment (T0), after treatment (T1), and 6 plus or minus 1 months of T1 (T2).

The study was approved by the Regional Ethics Committee of Marche (protocol ID 101/2024, number 3054) and conformed to the Declaration of Helsinki. All subjects gave their written informed consent to the use of clinical data for research purposes. INTENSO is registered with www.clinicaltrials.gov (NCT06695286). This study adheres to the STROBE Statement for cohort studies, and a completed checklist is provided in the Supplementary Material^22^.

### Data analysis

The sample size was calculated, considering that the NMSS score has a negligible chance of improving in subjects with PD over 6 months without additional interventions^51^. We assumed that the exposure to high-intensity training would improve the NMSS score by 20% (compared to the baseline condition) at the end of the training. We computed that a minimum sample size of 40 subjects was necessary to reject the null hypothesis that any change in NMSS score would be similar in HIT and LIT subgroups (with an alpha error of 0.05 and a beta error of 0.20).

We used descriptive statistics for demographic and clinical measures. Specifically, we reported MDS-UPDRS total and subtotals, NMSS total and subtotals, and FoG-Q total in the two subgroups. For the NMSS, we computed subtotals in the following domains (D): Autonomic (D1 – Cardiovascular including falls; D6 - Gastrointestinal tract; D7 - Urinary), Sleep/Fatigue (D2) and Cognition/Emotion (D3 - Mood/Cognition; D4 – Perceptual Problems Hallucination; D5 – Attention/Memory). We adopted a propensity-score matching to identify patients with similar age, disease duration, and Hoehn and Yahr scores and ensure proper comparability of HIT and LIT groups at baseline. We analyzed the within-group changes (time effect), at T1 or T2 follow-up with respect to T0, using the Wilcoxon rank test. We computed the inter-group comparisons (group effect) applying the Mann-Whitney test at each time point. We calculated the percentage changes of primary and secondary outcome measures at T1 and T2 with respect to T0. Δ delta: [(T1 or T2) score - (T0) score)/((T0) score)] x 100. We applied the simple regression analysis to check the relationship between Δ delta values and specific explanatory variables (i.e., age, disease duration, NMSS at T0, UPRDS part I at T0, MDS-UPDRS part II at T0). When appropriate, we applied a multiple regression analysis. Based on the application of Bonferroni correction for multiple comparisons, we set statistical significance at a *p*-value of <0.01. We used SAS StatView 5.0 for data analysis.

## Supplementary information

Supplementary Information

## Data Availability

The datasets used and/or analysed during the current study available from the corresponding author on reasonable request.
